# Metabolism of *β*-mannans by representative, understudied Bacillota species from the human colon

**DOI:** 10.1093/femsec/fiag063

**Published:** 2026-06-12

**Authors:** Galiana Lo, Sabina Leanti La Rosa, Bjørge Westereng, Phillip B Pope, Petra Louis, Carlos Sabater, Sylvia H Duncan

**Affiliations:** Gut Health Group, Rowett Institute, University of Aberdeen, Foresterhill, Aberdeen, Scotland AB25 2ZD, United Kingdom; Faculty of Biosciences, Norwegian University of Life Sciences, 1432 Aas, Norway; Faculty of Chemistry, Biotechnology and Food Science, Norwegian University of Life Sciences, 1432 Aas, Norway; Faculty of Biosciences, Norwegian University of Life Sciences, 1432 Aas, Norway; Faculty of Chemistry, Biotechnology and Food Science, Norwegian University of Life Sciences, 1432 Aas, Norway; Centre for Microbiome Research, Faculty of Health, School of Biomedical Sciences, Queensland University of Technology, Translational Research Institute, Woolloongabba, 4102, Australia; Gut Health Group, Rowett Institute, University of Aberdeen, Foresterhill, Aberdeen, Scotland AB25 2ZD, United Kingdom; Gut Health Group, Rowett Institute, University of Aberdeen, Foresterhill, Aberdeen, Scotland AB25 2ZD, United Kingdom; Gut Health Group, Rowett Institute, University of Aberdeen, Foresterhill, Aberdeen, Scotland AB25 2ZD, United Kingdom

**Keywords:** mannans, *Roseburia*, *Faecalibacterium*, *Coprococcus*, butyrate, prebiotics

## Abstract

The macronutrients in our diets including non-starch polysaccharides such as β-mannans, found in plant cell walls, can impact on human gut health. There is however a paucity of data regarding the ability of gut bacteria, in particular those belonging to the Bacillota (previously Firmicutes) phylum, to depolymerise and ferment β-mannans. In this study, we tested a total of 12 strains, including nine Bacillota, for their ability to metabolise and cross-feed on β-mannans. Three of the six butyrate-producing Bacillota strains, namely *Roseburia intestinalis* L1-82, *Roseburia faecis* M72/1, and *Coprococcus eutactus* ART55/1, were able to metabolise carob galactomannan, konjac glucomannan, and softwood spruce acetylated galactoglucomannan, which corresponded with their carbohydrate active enzyme profiles, whilst *Faecalibacterium prausnitzii* S3L/3 only grew well on β-mannan endo-mannanase digests. To investigate competition and microbial cross-feeding on β-mannans, growth assays were conducted with co-cultures of up to six strains belonging to both the Bacillota phylum and a Bacteroidetes β-mannan utilising strain, *Bacteroides ovatus* V975. All strains in the mixes were able to co-exist, including the non-mannan degrading butyrate producers, with butyrate being formed as one of the major fermentation products. These studies suggest that β-mannans may offer a notable prebiotic approach to promoting butyrate-producing bacteria and gut health.

## Teaser

Representative human gut butyrate producers belonging to the Bacillota phylum tested in this study were able to utilise carob (galactomannan), konjac (glucomannan), and spruce (acetylated galactoglucomannan) mannans as growth substrates.

## Introduction

The human gastrointestinal tract hosts a dense and complex microbial ecosystem (Sender et al. 2016), with most bacteria belonging to the Bacillota and Bacteroidetes phyla and lower numbers belonging to the Actinobacteria, Proteobacteria and Verrucomicrobia phyla (Tap et al. 2009, The Human Microbiome Project Consortium 2012, Duncan and Flint 2013). Key bacterial species belonging to the Bacteroidetes and Actinobacteria phyla are well known to possess enzyme systems that can degrade complex polysaccharides which host intestinal cells lack. Most of these bacteria are anaerobes that ferment dietary polysaccharides to mainly short chain fatty acids (SCFAs) (Flint et al. 2015), including acetate, propionate and butyrate. In particular, the latter helps to maintain the health of colonocytes (Ruemmele et al. 2003, Mukhopadhya and Louis 2025) and production may be promoted using prebiotic substrates. Prebiotics are defined as substrates that selectively stimulate members of the gut microbiota with health-conferring properties for the host (Ramirez-Farias et al. 2009, Macfarlane and Macfarlane 2011, Gibson et al. 2017). This includes stimulation of butyrate producers directly or indirectly through cross-feeding interactions (Rios-Covian et al. 2015, Gibson et al. 2017, Louis and Flint 2016).

Despite belonging to the dominant phylum, Bacillota species and their metabolism of complex dietary carbohydrates are however relatively understudied compared to those from the Bacteroidetes and Actinobacteria phyla. Saccharides, such as fructo-oligosaccharides and galacto-oligosaccharides, have traditionally been used as prebiotics to promote the growth of intestinal bacterial species including, in particular, certain lactobacilli and bifidobacteria species (Carlson et al. 2018). It is, however, important to note that species belonging to these genera mainly produce lactate and acetate from carbohydrate fermentation (Flint et al. 2015). Lactate accumulation in some cases may be associated with the severity of inflammatory bowel disease and other health conditions (Vernia et al. 1988, Hove et al. 1994, Louis et al. 2022). Additionally, *Bifidobacterium* and *Lactobacillus* spp. were reported to be increased in individuals with active inflammatory bowel disease while butyrate-producing Bacillota were decreased (Wang et al. 2014). This suggests that probiotic strains of bifidobacteria and lactobacilli may be beneficial but require careful consideration for individuals with gastrointestinal diseases. It also highlights the importance of conducting more studies on saccharides that promote butyrate producers, including members of the Bacillota phylum, *Faecalibacterium prausnitzii* and *Roseburia* spp., which have been gaining much interest as next-generation probiotic candidates (Brodmann et al. 2017, Duncan and Sabater 2025, Sabater et al. 2025).

**Figure 1:**
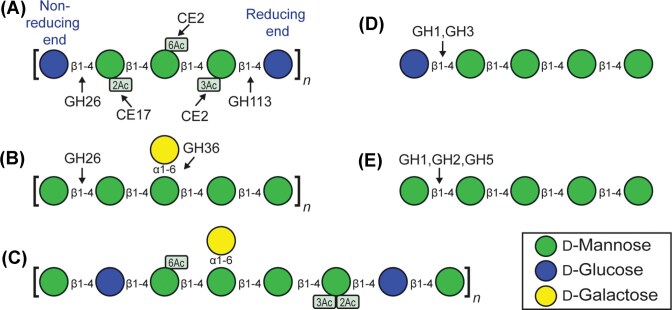
Structures of native (A) Konjac glucomannan (2Ac, 2-*O*-Acetyl; 3Ac, 3-*O*-Acetyl, 6Ac, 6-*O*-Acetyl, (B) Carob galactomannan, (C) Spruce galactoglucomannan. (D and E) show manno-oligosaccharides. β-Mannanases, found in the carbohydrate active enzyme (CAZyme) families, for example, glycoside hydrolase (GH) 26 and GH113, randomly cleave the β-1,4 linkages of the mannan main chain. α-Galactosidase (GH36) removes galactose side groups by targeting the α-1,6 bond. β-Mannosidase (GH1, GH2, GH5) breaks β-1,4 linkages between mannoses while carbohydrate esterases (CE) 2 and 17, remove acetyl groups. β-Glucosidase (GH1, GH3) cleaves the β-1,4 bond at non-reducing ends of manno-oligosaccharides. All information regarding enzyme degradation of mannans were from Moreira and Filho (2008). Enzyme activities found in each CAZyme family were from Lombard et al. (2014).

More recently, other saccharides including mannans and their respective oligosaccharides (Saville and Saville 2020) have also been considered to provide health benefits and β-mannans are mostly found in plants (Singh et al. 2018); these glycans can be insoluble or water-soluble. Examples of water-soluble β-mannans are glucomannan from konjac and galactomannan from carob (see Fig. 1). Forestry residues from softwoods may also hold promise as a sustainable source (La Rosa et al. 2019a) of mannan-type hemicelluloses, which comprise around 20% of their dry weight (Sjöström 1993). Mannans are mainly found in the form of acetylated galactoglucomannans in softwoods such as spruce (Timell 1967) and are structurally similar to the water-soluble β-mannans (see Fig. 1).

Plant β-mannans are widely distributed in the human diet because several, such as those found in konjac and carob, are used as food additives due to their stabilising and gel-forming properties (Singh et al. 2018). These complex polysaccharides may beneficially modulate the gut microbiota to promote health. For example, cellulase-treated konjac glucomannan has been shown to alleviate constipation and diarrhoea in inflammatory bowel disease patients (Suwannaporn et al. 2013). Moreover, spruce acetylated galactoglucomannan in addition to both carob galactomannan and konjac glucomannan can promote the growth of *R. intestinalis* which is a butyrate producer (La Rosa et al. 2019b). Similarly, digested galactoglucomannan restores colonic microbial diversity and provides chemo-preventive protection against colon cancer in rats (dos Santos Lima et al. 2024). Separately, it has been reported that some other butyrate-producing Bacillota from the human gut possess the ability to degrade these β-mannans (La Rosa et al. 2019b, Alessi et al. 2020). Bhattacharya et al. (2022) also investigated cross feeding between *B. adolescentis* ATCC 15703 and *R. hominis* A2-183 DSMZ 16839 using mannooligosaccharides (MOS) as substrates, revealing that these two bacterial strains could co-exist due to differential utilisation of MOS. Due to the complexity of the human gut microbiota, there are likely to be other bacterial strains with β-mannan degrading abilities that have not been investigated. It has however been reported that the growth of *Roseburia hominis* DSM 16839 and *Bifidobacteria adolescentis* ATCC 15703 were promoted on acetylated galactoglucomannan and carob galactomannan pre-treated with an endomannanase from *Bacteroides ovatus* (*Bo*Man26) (Bhattacharya et al. 2021). Potential cross-feeding interactions between non-butyrate-producing mannan degraders, for example dominant *Bacteroides* species (Bågenholm et al. 2017), and butyrate producers that are unable to metabolise mannans, therefore, warrants further investigation.

Here, we investigated the ability of representatives of the dominant phyla found in the human large intestine with a focus on several understudied Bacillota to metabolise water-soluble native plant β-mannans and β-mannan digests as well as cross-feeding interactions. We report on the superior ability of a number of dominant butyrate-producing Bacillota from the human colon to grow on β-mannans compared to species belonging to other bacterial phyla when tested in monoculture. Moreover, these butyrate producers also competed well within more complex bacterial combinations, suggesting that β-mannans may offer an important prebiotic approach to selectively promote butyrate-producing bacteria and, in turn, host health.

## Materials and methods

### Mannan substrates

The konjac (containing acetyl groups) and carob mannans in their native forms were purchased from Megazyme International (Wicklow, Ireland). The method used to prepare spruce-derived acetylated galactoglucomannan and the composition of the final product has been reported previously (Michalak et al. 2020a). The pre-hydrolysed forms of the carob, konjac and spruce β-mannans were generated using a recombinant GH26 from *Roseburia intestinalis* (*Ri*GH26) as described previously (Lindstad et al. 2021).

### Bacterial strains

Twelve representative strains of the dominant phyla present in the human colon were tested for their ability to utilise various β-mannans in this study. Nine strains were Bacillota and there was one representative each from the Bacteroidetes, Actinobacteria and Proteobacteria phyla (Table 1).

**Table 1:** Bacterial strains tested in this study.

Phylum	Family	Genus and species	Strain ID	Major fermentation products	Source	Prevalence in human gut metagenomes (n = 1000)^a^	Genome accession number	Supplier or reference
Firmicutes	Lachnospiraceae	*Anaerobutyricum soehngenii*	L2-7	B	Human	66.6%	GCA_900209925.1	Barcenilla et al. (2000)
	Lachnospiraceae	*Blautia hydrogenotrophica*	DSM 10 507	A	Human	61.0%	GCA_000157975.1	Bernalier et al. (1996)
	Lachnospiraceae	*Coprococcus eutactus*	ART55/1	B	Human	32.0%	GCA_000210595.1	Louis et al. (2004)
	Lachnospiraceae	*Eubacterium rectale*	A1-86	B	Human	75.5%	GCA_000209935.1	Barcenilla et al. (2000)
	Ruminococcaceae	*Eubacterium siraeum*	V10Sc8a	A	Human	34.0%	GCA_000210635.1	Duncan et al. (2016)
	Ruminococcaceae	*Faecalibacterium prausnitzii*	S3L/3	B	Human	78.7%	GCA_000209855.1	Louis et al. (2004)
	Lachnospiraceae	*Roseburia faecis*	M72/1	B	Human	76.9%	GCA_001406815.1	Louis et al. (2004)
	Lachnospiraceae	*Roseburia intestinalis*	L1-82	B	Human	75.1%	GCA_900537995.1	Barcenilla et al. (2000)
	Ruminococcaceae	*Ruminococcus bicirculans*	80/3	A	Human	44.2%	GCA_000723465.1	Wegmann et al. (2014)
Bacteroidetes	Bacteroidaceae	*Bacteroides ovatus*	V975	A, P, S	Human	76.6%	GCA_900095495.1	Gift from T. R. Whitehead (USDA)
Actinobacteria	Bifidobacteriaceae	*Bifidobacterium adolescentis*	L2-32	A, L	Human	76.1%	GCA_000154085.1	Duncan et al. (2004)
Proteobacteria	Enterobacteriaceae	*Escherichia coli*	K12 (DSM 498)	ND	Laboratory (James *et al*. 2001)	NA	GCA_000005845.2	DSMZ

a Protologger (Hitch et al. 2021).

Abbreviation: DSMZ: Deutsche Sammlung von Mikroorganismen und Zellkulturen (Braunschweig, Germany); A: Acetate; B: Butyrate; P: Propionate; L: Lactate; S: Succinate; ND: no data; NA: not applicable

### Assessment of bacterial growth and growth rates on mannans using a miniaturised microtitre plate assay

To assess the utilization of β-mannans for growth by representative human gut bacterial strains, three types of substrates, which were low viscosity (∼10 cSt) konjac acetylated glucomannan (60% mannose, 40% glucose), low viscosity (∼10 cSt) carob galactomannan (76% mannose, 24% galactose), and spruce acetylated galactoglucomannan, were used in the study. The *Roseburia intestinalis Ri*GH26-digested forms of these three mannans were included in the growth assays. Glucose was purchased from Fisher Scientific (New Hampshire, USA).

In preparation for the growth assays, the strains were inoculated into M2GSC medium (Miyazaki et al. 1997) under an atmosphere of CO_2_ and incubated at 37°C overnight. The assays were conducted in flat-bottomed Corning 96-well microplates (Sigma-Aldrich, St. Louis, MO, USA) in a MACS-VA500 anaerobic workstation (Don Whitley Scientific, Shipley, UK) with a gas phase of 80% N_2_, 10% H_2_, 10% CO_2_, using an Epoch 2 microplate spectrophotometer (BioTek, Vermont, USA). Sterile stock solutions (1%) of the substrates were prepared using basal M2 medium in place of distilled water. Konjac glucomannan and carob galactomannan were solubilised according to manufacturer’s instructions (Megazyme International, Wicklow, Ireland) with seven minutes of boiling to achieve sterility. Basal M2 medium was supplemented with 0.2% (w/v) of the substrates (final volume of 200 µl) and 5 µl of bacterial pre-cultures were inoculated into each well. There were three replicate wells for each bacterial strain. The inoculated plates were sealed with PCR plate seals (Bio-Rad, UK) to minimise evaporation. They were then incubated for 24–48 h at 37°C. OD readings were taken at 650 nm (OD_650_) over the time period of the experimental run, every 10 min (Gen5 Microplate Reader and Imaging Software) with orbital shaking for 10 s before a reading was taken. Basal M2 medium supplemented with glucose was included in every experimental run as a positive control to verify the viability of the strains. An exception was *Eubacterium siraeum* V10Sc8a, which does not utilize glucose as reported by Moore et al. (1976). Growth rates were determined as described previously during exponential growth (Pirt 1975).

### Cross-feeding interactions in mixed bacterial cultures when supplemented with native konjac mannan measuring growth in microtitre plates

Bacterial mixes involving five combinations of six bacterial strains with varying abilities to degrade konjac mannan were grown in 0.2% konjac (w/v) supplemented basal M2 medium to assess growth and metabolites produced. The bacterial mixes are shown in Table 2. The experiments were set up similarly to what has been described above and 5 µl of pre-cultures were inoculated singly or as bacterial mixtures into a total volume of 200 µl. The OD_650_ values of the pre-cultures were measured before inoculation and the final volume for each well was 200 µl. Optical density readings to assess growth were from a minimum of three replicates. Basal M2 media with or without glucose were included in each plate. The growth of each individual strain on the three media tested were also assessed on the same plate with the bacterial mixes.

**Table 2:** Composition of bacterial mixes used in mixed culture growth assays.

2-strain mix	3-strain mix	4-strain mix	5-strain mix	6-strain mix
*Roseburia faecis* M72/1^a^	*Roseburia faecis* M72/1^a^	*Roseburia faecis* M72/1^a^	*Roseburia faecis* M72/1^a^	*Roseburia faecis* M72/1^a^
*Faecalibacterium prausnitzii* S3L/3^a^	*Faecalibacterium prausnitzii* S3L/3^a^	*Faecalibacterium prausnitzii* S3L/3^a^	*Faecalibacterium prausnitzii* S3L/3^a^	*Faecalibacterium prausnitzii* S3L/3^a^
	*Bacteroides ovatus* V975	*Bacteroides ovatus* V975	*Bacteroides ovatus* V975	*Bacteroides ovatus* V975
		*Coprococcus eutactus* ART55/1^a^	*Coprococcus eutactus* ART55/1^a^	*Coprococcus eutactus* ART55/1^a^
			*Anaerobutyricum soehngenii* L2-7^a^	*Anaerobutyricum soehngenii* L2-7^a^
				*Blautia hydrogenotrophica* DSM 10 507

a Denotes butyrate producing strains.

### DNA extractions

After the mixed culture experiments were completed, cell pellets were obtained from pooled samples of triplicate wells for each condition tested (600 µl) and bacterial inocula (600 µl). The cell pellets were resuspended in buffers from the FastDNA SPIN Kit for Soil (MP Biomedicals) and stored at −70°C until DNA extraction. DNA extraction was performed using the FastDNA SPIN Kit for Soil (MP Biomedicals) following manufacturer’s instructions.

### Quantitative PCR

The extracted DNA from the mixed culture experiments were analysed using quantitative PCR described by Chung et al. (2016) with some modifications. Herring sperm (HS) DNA (5 ng/ml) (Promega, Madison, WI, USA) was used to dilute the samples and 16S PCR products of bacterial strains (*Roseburia faecis* M72/1, *Faecalibacterium prausnitzii* S3L/3*, Bacteroides ovatus* V975, *Coprococcus eutactus* ART55/1, *Anaerobutyricum soehngenii* L2-7, *Blautia hydrogenotrophica* DSM 10507), which were used to obtain a dilution series for standard curves. A master-mix containing iTaq Universal SYBR Green Supermix (Bio-Rad, Hemel Hempstead, UK), 10 µM primer pairs (500 nM final concentration) and molecular biology grade water was prepared. The master-mix (8 µl) together with 2 µl of either a diluted sample (1 ng/ µl) or standard, giving a total volume of 10 µl, was distributed into the wells of 384-well plates. The plates were then sealed with optical seals (Bio-Rad). The Bio-Rad CFX384 Real-Time System was used for the amplification of samples and standards using the protocol described by Chung et al. (2016) with annealing temperatures changed to that which is specific for each primer set (Table 3). The reactions were performed in triplicates and the number of 16S rRNA gene copies per mL of culture for each bacterial strain in the mixes tested were determined using information from the standard curves. Negative controls containing only HS DNA were used to determine the detection limit.

**Table 3:** Primers used for real-time qPCR.

16S target	Primer	Sequence (5′ to 3′)	Annealing temperature (°C)	Reference
Universal (Uni)	UniF	GTGSTGCAYGGYYGTCGTCA	60	Fuller et al. (2007)
	UniR	ACGTCRTCCMCNCCTTCCTC		
*Eubacterium hallii* ^a^ (Ehal)	EhalF	GCGTAGGTGGCAGTGCAA	60	Reichardt et al. (2018)
	EhalR	GCACCGRAGCCTATACGG		
*Blautia* spp.	BlautiaF2	CGCGTGAAGGAAGAAGTATC	63	Reichardt et al. (2018)
	BlautiaR2	GAGCCTCAACGTCAGTTACC		
*Coprococcus eutactus*	CeutFext/L2-50Fext	TTCCAGTAGCCAGCAGTMAGAT	63	Reichardt et al. (2018)
	CeutR	CAATCCGAACTGAGACAGCC		
*Faecalibacterium prausnitzii* (Fprau)	FprauF	TGAGGAACCTGCCTCAAAGA	63	Lindstad et al. (2021)
	FprauR	GACGCGAGGCCATCTCA		
*Roseburia* (Rrec1)	RrecF	GCGGTRCGGCAAGTCTGA	63	Ramirez-Farias et al. (2009)
	Rrec630mR	CCTCCGACACTCTAGTMCGAC		
*Bacteroides* spp.	g-Bfra-R-Fmod	GCTCAACCKTAAAATTGCAGTTG	63	Reichardt et al. (2018)
	Bac708Rmod	GCAATCGGRGTTCTTCGTG		

a These primers were used to detect *Anaerobutyricum soehngenii* L2-7, previously *Eubacterium hallii* (Shetty et al. 2018).

### Short chain fatty acid (SCFA) analysis

SCFA profiles were determined using capillary gas chromatography. Cultures from the microplate experiments (stored at −20°C) were pooled and aliquots of the pooled samples were analyzed in triplicate to obtain three technical replicates. Samples were processed according to the method previously described by Richardson et al. (1989). Briefly, the SCFAs were extracted by first acidifying the samples with 500 µl concentrated hydrochloric acid and then solubilising the SCFAs in diethyl ether (3 ml). For each sample. the diethyl ether with the extracted SCFAs (800 µl) were dispensed into a screw cap vial with N-tert-butyldimethylsilyl-N-methyltrifluoroacetamide (MTBSTFA) (100 µl) added and placed in a heating block at 80°C for 20 mins. The MTBSTFA-derivatized ether layers from the samples were then analysed using a Hewlett–Packard 6890 Gas Chromatograph with autosampler, fitted with a HP-1 methyl siloxane-coated capillary column (Agilent Technologies, Santa Clara, CA, USA). The carrier gas used was helium. Concentrations of the SCFAs were determined by obtaining response factors relative to the internal standard (50 µl)– 2-ethylbutyric acid, an acid not produced from bacterial fermentation of the tested substrates. 0.5 mM was the lower detection limit for changes in SCFA concentrations.

### Carbohydrate active enzymes (CAZymes) encoded within genomes of the tested strains

To gain a better understanding of mannan degradation, CAZymes potentially involved in β-mannan utilisation for the strains under study were organised into CAZyme profiles using genomic information. The genomic FASTA files for the bacterial strains were obtained from NCBI Assembly (https://www.ncbi.nlm.nih.gov/assembly) (Kitts et al. 2016) (Table 1) and ran through “run_dbcan” pipeline (dbCAN version 3) (Zhang et al. 2018), which maps the genome sequences against the CAZy database (http://www.cazy.org/ last accessed: 18/12/2025). This is an expert-curated database of profile hidden Markov models representing the signature domains of CAZyme families (GH, glycoside hydrolase; CE, carbohydrate esterase; CBM, carbohydrate-binding module). To ensure the quality of the data generated, only enzyme domains showing coverage values higher than 0.8 were chosen. A list of CAZymes known to be active on the β-mannan backbone and side chains was established by manually selecting and curating info from the CAZy database. For families known to include several specificities, the dbCAN3 annotations were cross-checked with gene annotations. Specifically, if a protein was annotated to be a GH2 by dbCAN3, the annotation for the gene product obtained from RAST (Aziz et al. 2008) was then checked to assess if it was a beta-mannosidase. Bacterial strains were grouped according to the CAZymes domains found in their genomes through hierarchical clustering considering a Euclidean distance metric. Then, a heatmap plot illustrating the presence and absence of different β-mannan-degrading CAZymes was generated using R (v.4.4.1).

### Metabolic and molecular modelling

Once CAZymes acting on β-mannan were annotated, potential metabolic interactions between microbial strains included in mixed bacterial cultures (*A. soehngenii* L2-7, *B. hydrogenotrophica* DSM 10507, *B. ovatus* V975, *C. eutactus* ART55/1, *F. prausnitzii* S3L/3, and *R. faecis* M72/1) were calculated. For this purpose, bacterial genomes were annotated using Prokka (v1.14.6) to generate standard Genbank files (.gbk) files that were used as input for metage2metabo (v1.5.0) software (Belcour et al. 2020, Calvete-Torre et al. 2023). This pipeline allows the reconstruction of genome-scale metabolic network (GSMN) of mannan-degraders. To simulate microbial metabolic interactions in the presence of mannan, a “seeds” file containing different nutrients present in M2 medium, in addition to different mannan structures (β-1,4-linked mannan, glucomannan, galactomannan and galactoglucomannan).

Enzyme affinity of β-mannan-degrading CAZymes annotated and mannan backbone was investigated through molecular docking. For this purpose, 3D conformations of mannanase and mannosidase CAZymes were generated using LocalColabFold v.1.5.5 (Mirdita et al. 2022) and a representative β-mannan tetrasaccharide was built on GLYCAM Carbohydrate Builder (Grant et al. 2025). Active site prediction of CAZymes and molecular docking were performed using p2rank v.2.4.1 (Krivák and Hoksza 2018) and Vina-Carb v.1.0 (Nivedha et al. 2016) software, respectively.

## Results

Lachnospiraceae and Ruminococcaceae strains belonging to the dominant, yet understudied Bacillota phylum were compared to strains of other phyla found in the human colon, namely a Bacteroidetes representative, *B. ovatus* previously reported to use β-mannans (Bågenholm et al. 2017) (Table 1). In addition, representative strains of the less dominant phyla, Actinobacteria and Proteobacteria, were included to assess their potential to metabolise β-mannans. This was done by measuring the growth of the bacterial strains on three different β-mannans and assessing the presence of genes encoding enzymes for β-mannan breakdown in their genomes using genome mining and information obtained from the CAZy database (see materials and methods).

### Growth of bacterial strains on β-mannans

**Figure 2:**
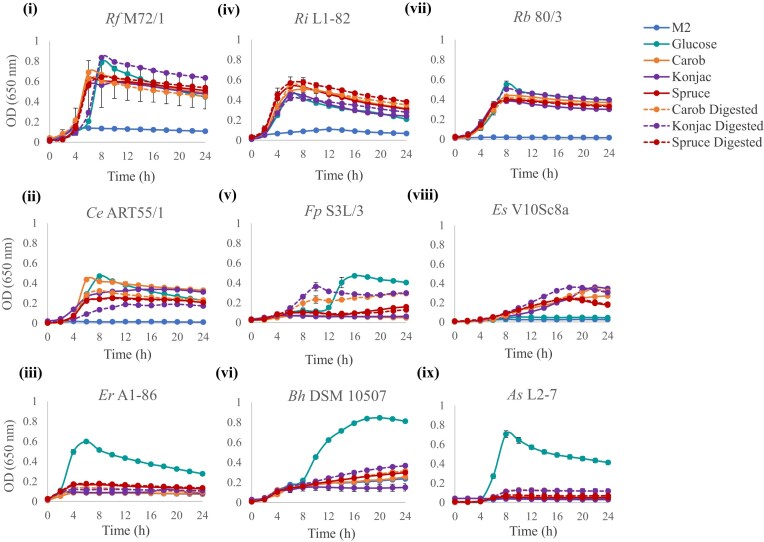
Growth curves plotted using mean optical densities (triplicate wells) with standard deviations from microtitre plate assays using representatives from the Firmicutes phylum on native konjac, carob and spruce mannans as well as their glycoside hydrolase (GH)-26 treated forms. The substrates were provided at a concentration of 0.2% (w/v). Rf: *R. faecis*; Ri: *R. intestinalis*; As: *A. soehngenii*; Ce: *C. eutactus*; Fp: *F. prausnitzii*; Rb: *R. bicirculans*; Er: *E. rectale*; Bh: *B. hydrogenotrophica*; Es: *E. siraeum*.

**Figure 3:**
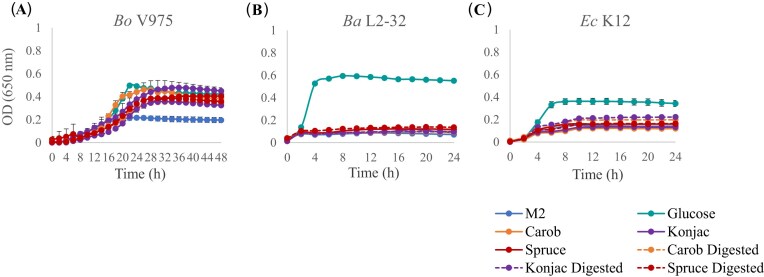
Growth curves plotted using mean optical densities (triplicate wells) with standard deviations from microtitre plate assays using representatives from the Bacteroidetes(A), Actinobacteria (B) and Proteobacteria (C) phyla on native konjac, carob and spruce mannans as well as their glycoside hydrolase (GH)-26 treated forms provided at a concentration of 0.2% (w/v). Optical densities measured at 650 nm that were less than 0.15 were considered as limited growth. Bo: *B. ovatus;* Ba: *B. adolescentis;* Ec: *E. coli*.

The Bacillota strains, *Anaerobutyricum soehngenii* L2-7, *Blautia hydrogenotrophica* DSM 10507, *C. eutactus* ART55/1, *Eubacterium rectale* A1-86, *E. siraeum* V10Sc8a, *Faecalibacterium prausnitzii* S3L/3, *Roseburia faecis* M72/1, *R. intestinalis* L1-82, and *R. bicirculans* 80/3, in addition to other phyla representatives, *B. ovatu*s V975 (Bacteroidetes), *B. adolescentis* L2-32 (Actinobacteria) and *E. coli* K12 (Proteobacteria) were assessed for their ability to utilise konjac-, carob-, and spruce mannan together with their RiGH26-digested forms. Growth on peptides in the basal medium was reported (Fig. 2). The growth rates and maximum optical densities at 650 nm (OD_650_) achieved by the tested strains are shown in Table 4. The *B. ovatus* strain V975 was able to utilise peptides in the basal M2 medium for growth and achieved a maximum OD_650_ value of 0.22 ± 0.02 (Fig. 3). Any additional increase in OD_650_ when B. ovatus V975 was supplied with the mannan substrates was, therefore, interpreted as growth on the mannans, in addition to growth supported by the peptides present in the basal medium. It is to be noted that while growth on basal M2 medium was considered during data interpretation, OD_650_ values in Table 4 were reported as measured without subtraction for this strain. *Eubacterium rectale* A1-86, *B. hydrogenotrophica* DSM 10507, *A. soehngenii* L2-7, *B. adolescentis* L2-32, and *E. coli* K12 were excluded from Table 4 as the OD_650_ values obtained from growth on the mannan substrates were less than 0.15 (Figs 2 and 3). This is likely due to these strains lacking many or most of the β-mannan degrading enzymes (the distribution of mannan-degrading enzymes in these bacteria is discussed in the next sections). Growth rates were not calculated for substrates where maximum OD_650_ values were less than 0.15.

**Table 4:** Growth rates (h^−1^) of bacteria that were able to utilise mannans, with maximum optical densities (650 nm) ± std dev shown in brackets.

Substrates	*Rf* M72/1^a^	*Ri* L1-82^a^	*Ce* ART55/1^a^	*Fp* S3L/3^a^	*Rb* 80/3	*Es* V10Sc8a	*Bo* V975^c^
Basal (M2)	ND (0.14 ± 0.00)	ND (0.11 ± 0.01)	ND (0.02 ± 0.01)	ND (0.07 ± 0.01)	ND (0.02 ± 0.00)	ND (0.03 ± 0.00)	0.59 ± 0.22 (0.22 ± 0.02)
Glucose	0.51 ± 0.01 (0.79 ± 0.01)	0.73 ± 0.02 (0.47 ± 0.02)	0.93 ± 0.06 (0.47 ± 0.01)	0.20 ± 0.03 (0.49 ± 0.02)	0.49 ± 0.04 (0.55 ± 0.03)	ND (0.05 ± 0.00)	0.44 ± 0.16 (0.50 ± 0.02)
Konjac	0.72 ± 0.34 (0.59 ± 0.16)	1.00 ± 0.12 (0.45 ± 0.01)	0.77 ± 0.16 (0.35 ± 0.06)	ND (0.07 ± 0.01)	0.68 ± 0.00 (0.38 ± 0.02)	0.51 ± 0.05 (0.36 ± 0.01)	0.55 ± 0.22 (0.49 ± 0.04)
GH26- treated konjac	0.63 ± 0.11 (0.83 ± 0.01)	0.80 ± 0.06 (0.42 ± 0.02)	0.42^b^ (0.19^b^)	0.35 ± 0.02 (0.36 ± 0.04)	0.56 ± 0.03 (0.50 ± 0.01)	0.45 ± 0.08 (0.36 ± 0.00)	0.59 ± 0.12 (0.36 ± 0.00)
Carob	0.74 ± 0.14 (0.69 ± 0.02)	0.93 ± 0.02 (0.51 ± 0.03)	1.12 ± 0.07 (0.44±0.04)	ND (0.07 ± 0.01)	0.75 ± 0.07 (0.44 ± 0.02)	0.48 ± 0.01 (0.27 ± 0.01)	0.40 ± 0.08 (0.50 ± 0.01)
GH26-treated carob	0.63 ± 0.04 (0.63 ± 0.01)	0.89 ± 0.06 (0.55 ± 0.02)	1.04 ± 0.05 (0.32±0.01)	0.30 ± 0.02 (0.29 ± 0.01)	0.58 ± 0.03 (0.41 ± 0.01)	0.49 ± 0.04 (0.34 ± 0.01)	0.63 ± 0.03 (0.36 ± 0.00)
Spruce	0.61 ± 0.08 (0.66 ± 0.00)	0.88 ± 0.10 (0.54 ± 0.09)	0.92 ± 0.14 (0.25 ± 0.01)	0.36 ± 0.01 (0.16 ± 0.00)	0.60 ± 0.02 (0.40 ± 0.02)	0.59 ± 0.16 (0.23 ± 0.01)	0.36 ± 0.15 (0.41 ± 0.00)
GH26-treated spruce	0.65 ± 0.02 (0.68 ± 0.00)	0.96 ± 0.01 (0.53 ± 0.01)	0.98 ± 0.05 (0.25 ± 0.00)	ND (0.13 ± 0.01)	0.56 ± 0.02 (0.40 ± 0.00)	0.39 ± 0.04 (0.24 ± 0.00)	0.59 ± 0.21 (0.39 ± 0.00)

a Butyrate producers

b Duplicates

c Taking into account growth on basal M2 medium, its growth on GH26-treated konjac, GH26-treated carob, spruce and GH26-treated spruce was minimal.

ND: not done as growth rates were not calculated for substrates with maximum OD_650_ less than 0.15.

Maximum optical density values at 650 nm were from Figs 2 and 3.

Growth studies revealed that the Bacillota strains, *R. faecis* M72/1, *R. intestinalis* L1-82, *R. bicirculans* 80/3, and *E. siraeum* V10Sc8a were able to utilize all three of the native β-mannans and their pre-digested forms (Fig. 2 and Table 4). *C. eutactus* ART55/1 grew minimally on GH26-treated konjac but was able to utilise the other substrates tested (Table 4).

*Faecalibacterium prausnitzii* S3L/3, on the other hand, was only able to utilise the GH26-treated forms of konjac and carob mannan with very minimal growth on the native and pre-treated spruce acetylated galactoglucomannan (Table 4). This demonstrates the need for bacteria with extracellular endo-β-1,4-mannanase (GH26) to be present in order for this strain to utilise mannans with high degree of polymerization, even though it possesses most of the other CAZymes commonly involved in β-mannan degradation (Fig. 5). In summary, of the six butyrate producers, only three of the strains, namely *R. faecis* M72/1, *R. intestinalis* L1-82, and *C. eutactus* ART55/1, were able to utilise the native β-mannans.

Statistical analyses were performed comparing differences in maximum OD_650_ values and growth rates between native β-mannans and their pre-treated counterparts. The results revealed no significant differences in maximum OD_650_ values and growth rates when *R. intestinalis* L1-82 was cultured on different sources of β-mannans as well as on β-mannans with or without pre-treatment (OD_650_ values: *P* = 0.001; Welch’s ANOVA, *P* > 0.05; independent *t*-test, growth rate: *P* = 0.063; Welch’s ANOVA). For *R. bicirculans* 80/3, OD_650_ values were only significantly higher on the pre-treated konjac glucomannan compared to its native form (*P* < 0.001; one-way ANOVA, *P* < 0.001; Tukey’s post hoc test). Furthermore, growth rates were significantly higher on the native forms of konjac glucomannan and carob galactomannan than on their GH26-digested forms (*P* = 0.002; Welch’s ANOVA, *P* = 0.024, *P* = 0.036, respectively; independent *t*-test). *R. faecis* M72/1 achieved significantly higher maximum OD_650_ values on the pre-treated konjac glucomannan and acetylated spruce galactoglucomannan compared to their respective native forms (*P* < 0.001; Welch’s ANOVA, *P* = 0.002, *P* = 0.015, respectively; independent *t*-test). In addition, growth rates on the mannan substrates were not found to be significantly different for this strain (*P* = 0.804; Welch’s ANOVA). For *E. siraeum* V10Sc8a, among the three native mannans, maximum OD_650_ values were significantly higher on konjac glucomannan [*P* < 0.001; Welch’s ANOVA, *P* = 0.004 (carob galactomannan), *P* < 0.001 (acetylated spruce galactoglucomannan); independent *t*-test]. The maximum OD_650_ value achieved on native carob galactomannan compared to GH26-digested carob galactomannan was significantly lower (*P* < 0.001; Welch’s ANOVA, *P* = 0.001; independent *t*-test). The growth rates, however, were not significantly different (*P =* 0.212; Welch’s ANOVA).

Other than Bacillota species, other genera, including *Bacteroides* species can have a role in mannan metabolism. *Bacteroides ovatus* strain V975 was able to utilise peptides in the basal M2 medium for growth and achieved a maximum OD_650_ value of 0.22 ± 0.02 (Fig. 3). Any additional increase in OD_650_ when *B. ovatus* V975 was supplied with the mannan substrates was, therefore, interpreted as growth on the mannans in addition to the peptides present in the basal medium. It is to be noted that 0.22 was not subtracted from the maximum OD_650_ values presented in Table 4 for the mannan substrates. The maximum OD_650_ values for the mannan substrates were then compared to that achieved on glucose (0.50 ± 0.02). The maximum OD_650_ values on native konjac (0.49 ± 0.04) and carob (0.50 ± 0.01) were similar to that on glucose, indicating the ability of *B. ovatus* V975 to grow on both native konjac and carob mannan sources. In contrast, *B. ovatus* V975 achieved maximum OD_650_ values of 0.36 ± 0.00 on the pre-digested konjac and carob mannans (Table 4) and in addition achieved maximum OD_650_ values of 0.41 ± 0.00 on native spruce acetylated galactoglucomannan and 0.39 ± 0.00 on its GH26-treated form. These values taken together with its growth on basal M2 medium (0.22 ± 0.02) suggest the ability of *B. ovatus* V975 to grow on these substrates but perhaps to a lesser extent compared to glucose, native konjac and native carob.

*Bifidobacteria adolescentis* L2-32 grew well on glucose while *E. coli* K12 grew on basal M2 medium (which contains peptides) with or without glucose. Both strains, however, achieved lower maximum OD_650_ values on the tested mannan substrates (Fig. 3) compared to glucose, with values being less than 0.15 (after taking into account growth on basal M2 for *E. coli* K12). The maximum OD_650_ value for *E. coli* K12 cultured in anaerobic basal M2 was, however, relatively lower compared to the other peptide utilisers tested in this study (*B. ovatus* V975 and *B. hydrogenotrophica* DSM 10507) (Table 4).

Growth rates on the substrates for strains that were able to utilise β-mannans were generally slightly higher compared to glucose (Table 4). Further, the maximum OD_650_ values achieved when grown on native carob galactomannan and glucose were very similar for *R. intestinalis* L1-82, *C. eutactus* ART55/1 and *B. ovatus* V975 (Table 4). The abilities to utilize konjac glucomannan was then compared between the butyrate-producing mannan-degrading Bacillota (Table 4 excluding *F. prausnitzii*) and *B. ovatus* V975. Results showed that the three Bacillota strains achieved significantly higher OD_650_ values than *B. ovatus* V975 [*P <* 0.001; Welch’s ANOVA, *P* = 0.001 (compared to ART55/1), *P* < 0.001 (compared to M72/1 and L1-82); independent *t*-test].

### Growth of bacterial strains when co-cultured on konjac glucomannan

Several butyrate-producing mannan degraders were identified from the previous set of experiments and one in particular, *F. prausnitzii* S3L/3, showed the potential to utilise β-mannans when first pre-treated with a GH26 endo-mannanase from *R. intestinalis* L1-82. To test if the pre-digesting step could be fulfilled by other bacteria possessing GH26, growth assays involving five bacterial mixes (Table 2) cultured on konjac glucomannan were performed. The possible competition and cross-feeding interactions present among bacteria when cultured together were also examined.

First, *F. prausnitzii* S3L/3 was co-cultured with *R. faecis* M72/1, then *B. ovatus* V975 was added to the mix, followed by *C. eutactus* ART55/1, which is a mannan-degrading butyrate producer, followed by *A. soehngenii* L2-7 that converts lactate to butyrate, and finally, *B. hydrogenotrophica* DSM 10507, which is an acetogen that uses formate.

**Figure 4:**
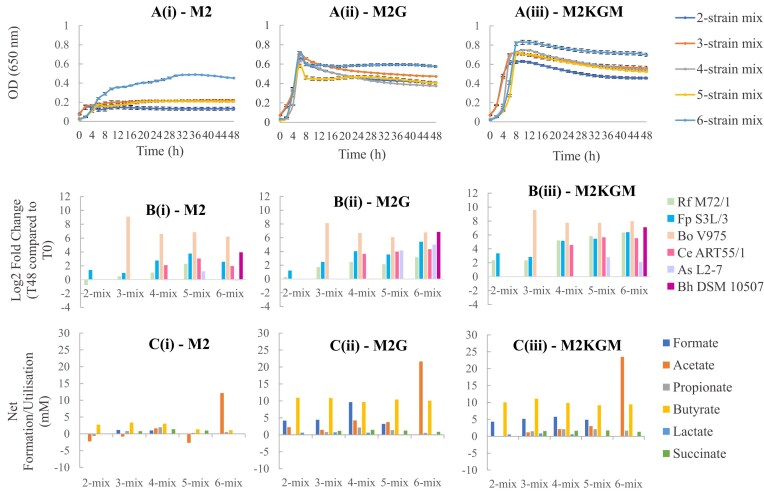
From top to bottom, the panels show the following: growth curves (OD_650_) (A), fold growth changes (log2) (B), and SCFA profiles (C) of the six bacterial mixes grown in basal M2 media (with no added sugars, containing peptides): A–C(i), M2 media supplemented with 0.2% glucose (M2G): A–C(ii) andl M2 supplemented with 0.2% konjac glucomannan (M2KGM): A–C(iii). Growth curves were plotted using the mean optical density values (OD 650 nm) from a minimum of three wells with standard deviation error bars. Bar charts in the middle row (row B) show fold growth changes by determining the number of 16S rRNA gene copies/mL of culture using qPCR, at the start (0 h) and end (48 h) of experimental runs which were expressed as T48/T0 fold changes and then Log_2_-transformed. Values were means from pooled samples of three biological replicates. Means of SCFA concentrations (mM) from pooled biological replicates are also plotted (row C). Standard deviations of the technical triplicates are in Supplemental Tables S3 and S4. SCFA concentrations measured in uninoculated M2, M2G, and M2KGM media were subtracted from the values. The 2-strain mix contained: *R. faecis, F. prausnitzii*; 3-strain mix: *R. faecis, F. prausnitzii, B. ovatus*; 4-strain mix: *R. faecis, F. prausnitzii, B. ovatus, C. eutactus*; 5-strain mix: *R. faecis, F. prausnitzii, B. ovatus, C. eutactus, A. soehngenii*; 6-strain mix: *R. faecis, F. prausnitzii, B. ovatus, C. eutactus, A. soehngenii, B. hydrogenotrophica.*

In the 2-strain mix, butyrate concentrations were higher (10 mM) (Fig. 4) than when the two strains were cultured separately (8 mM and 2 mM for *R. faecis* M72/1 and *F. prausnitzii* S3L/3, respectively, as shown in Fig. S1B). Further, konjac promoted better growth, as shown from a greater Log2 fold change, of both *R. faecis* M72/1 and *F. prausnitzii* S3L/3 compared to growth on glucose (Fig. 4). When *B. ovatus* V975 was added, *R. faecis* M72/1 and *F. prausnitzii* S3L/3 were still able to grow but growth for the latter was slightly lower compared to the 2-strain mix condition [Fig. 4B(iii)]. The positive fold changes of the bacteria when the 4- strain mix was cultured on M2 basal media showed that they were likely cross-feeding on metabolites [Fig. 4B(i)], as only *B. ovatus* V975 was able to utilise peptides within this strain combination (Fig. S1A). The fold change of each strain increased when supplied with glucose, with growth better on the konjac substrate [Fig. 4B(ii) and (iii)]. The same observation could be made for all strains in the 5-strain mix except for *A. soehngenii* L2-7 whose 16S rRNA copy numbers decreased when grown on konjac [Fig. 4B(iii)], which is not surprising as it fails to degrade mannan. The 4-strain mix did not contain lactate utilisers (Table 2), thus the concentration of lactate detected when the strains when cultured on glucose (0.63 mM) and konjac (0.57 mM) reflected net production. Since lactate production was minimal (as seen from the 4-strain mix), this may not contribute to the growth of *A. soehngenii* L2-7 in the 5-strain mix and the increased fold change when cultured on konjac compared to basal M2, therefore, may suggest utilization of β-mannan breakdown products from β-mannan degraders in the mix [Fig. 4B(i) and (ii)]. In the 6-strain mix, acetate concentrations were higher while butyrate concentrations were comparable to that of the 5-strain mix [Fig. 4C(iii)]. No formate was detected [Fig. 4C(ii) and (iii)], which is most likely used by *B. hydrogenotrophica* DSM 10507 to form acetate, although it was unable to degrade mannans [Fig. 2(vi)]. Overall, butyrate levels were comparable between the strain mixes when comparing growth on glucose and konjac. The fold change of the butyrate producers *R. faecis* M72/1, *F. prausnitzii* S3L/3, and *C. eutactus* ART55/1, however, was higher when provided with konjac compared to glucose.

### Distribution of β-mannan-degrading enzymes among the tested bacterial strains and mannan utilisation ability predictions

**Figure 5:**
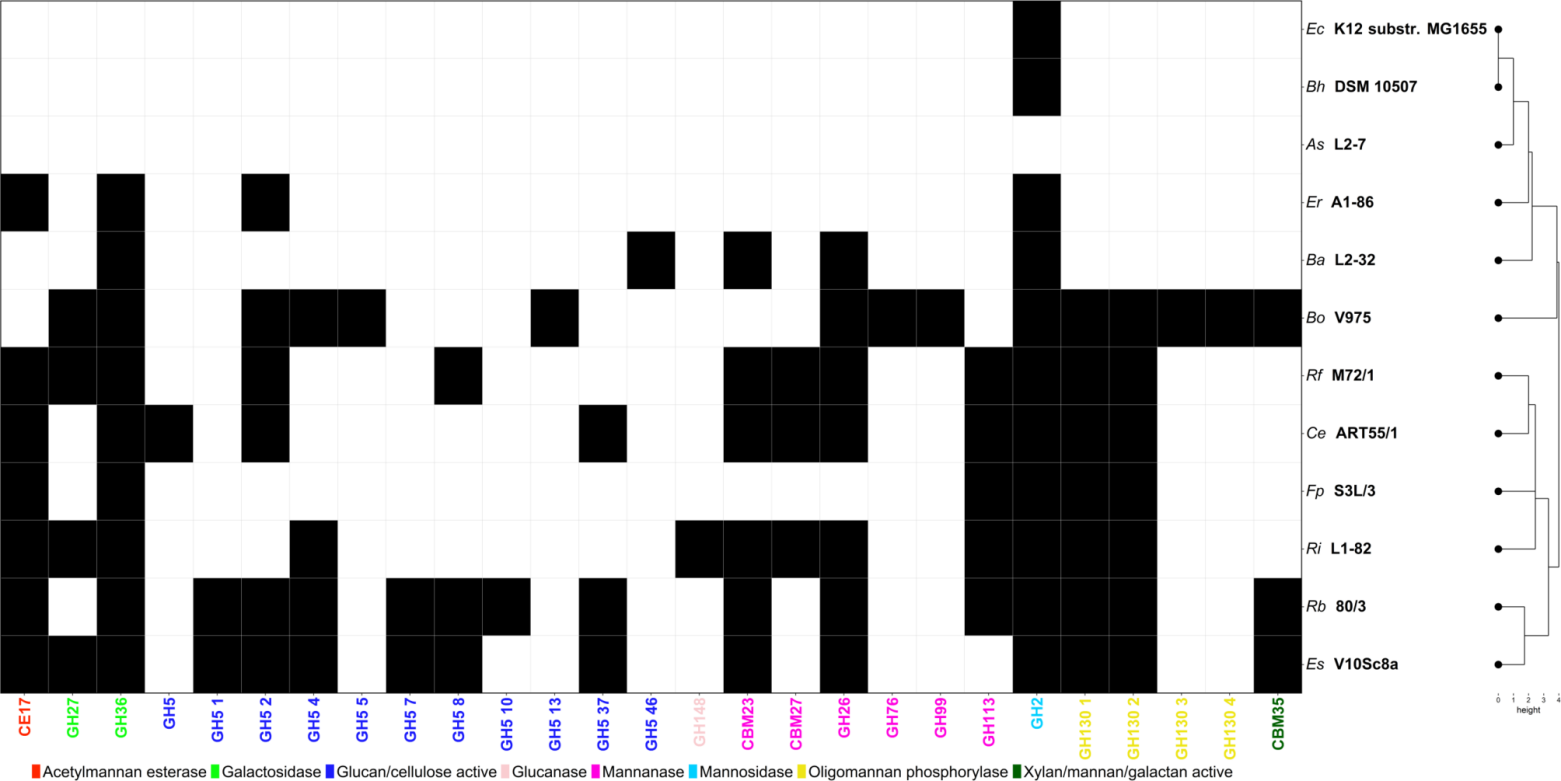
Heatmap showing the presence of different enzymes acting on mannan, glucomannan, galactomannan, and galactoglucomannan structures in the genome sequences of bacterial strains tested in this study. Black and white cells indicate presence/absence of each CAZyme, respectively. Codes corresponding to the CAZymes Database family of each enzyme have been assigned. Strains were grouped according to the CAZyme domains found in their genomes through hierarchical clustering considering a Euclidean distance metric.

Metabolic capabilities of microbial isolates were investigated *in silico* to gain a better understanding of the enzyme activities involved in mannan degradation (Fig. 5). Several enzymes involved in the degradation of β-mannans have been reported to belong to the glycoside hydrolase (GH) families 2, 5, 26, 27, 36, and 130, and carbohydrate esterase (CE) family 17. Figure 5 illustrates several microbial CAZymes needed to break down mannans from konjac, carob and spruce. The presence/absence of these CAZymes indicates the ability of each strain to degrade β-mannan backbone and other mannan domains involving galactose and glucose monomers.

Mannanases and mannosidases acting on β-1,4-linked mannan backbone were annotated in the majority of strains although *A. soehngenii* L2-7, *B. hydrogenotrophica* DSM 10507, and *E. rectale* A1-86 showed the lowest range of these activities. Similarly, CAZymes that hydrolyse galactose residues from galactomannan and galactoglucomannan chains were annotated in all strains with the exception of *A. soehngenii* L2-7, *B. hydrogenotrophica* RSM 10507, and *E. coli* K12. A wide range of glycosidases and glucanases comprising GH5 subfamilies were found in *E. siraeum* V10Sc8a and *R. bicirculans* 80/3 highlighting the potential of these strains to metabolise glucomannan and galactoglucomannan structures.

Possessing either GH26 or GH5 subfamilies indicates the ability to degrade native β-mannans to manno-oligosaccharides (β-MOS). Bacterial strains without GH26 and GH5 may be deemed to be unable to utilise the three native mannans. Further metabolisation of β-MOS occurs if GH130s (present in *B. ovatus* V975, *R. faecis* M72/1, *C. eutactus* ART55/1, *F. prausnitzii* S3/L, *R. intestinalis* L1-82, Rb 80/3, *E. siraeum* V10Sc8a), GH36 (present in all strains with the exception of *E. coli* K12, *B. hydrogenotrophica* DSM 10507 and *A. soehngenii* L2-7), and mannosidases such as GH113s (present in *R. faecis* M72/1, *C. eutactus* ART55/1, *F. prausnitzii* S3/L, *R. intestinalis* L1-82, Rb 80/3) or GH2s (present in all strains with the exception of *A. soehngenii* L2-7) are encoded by their genome.

On the other hand, acetylmannan esterases including CE17 acting on acetyl side groups from the β-mannan backbone were identified in the genome sequences of different genera including *Coprococcus, Eubacterium, Faecalibacterium, Roseburia*, and *Ruminococcus* (Fig. 5). It should be noted that *E. coli* K12 lacked most of the CAZymes required for β-mannan metabolism including endo-β-1,4-mannanases and several GH5 subfamilies.

Several enzyme activities are required for utilisation of complex mannans. Carbohydrate esterases are likely to be important for konjac glucomannan and spruce galactoglucomannan degradation as they are acetylated. Moreover, α-galactosidases belonging to the GH36 family are crucial for carob galactomannan metabolism due to the presence of galactose side chains. For example, *R. faecis* M72/1 possessed all of the CAZymes involved in the degradation of all three native mannans and their GH26-treated forms. This bacterium has the metabolic potential to degrade all of the tested mannan substrates.

**Figure 6:**
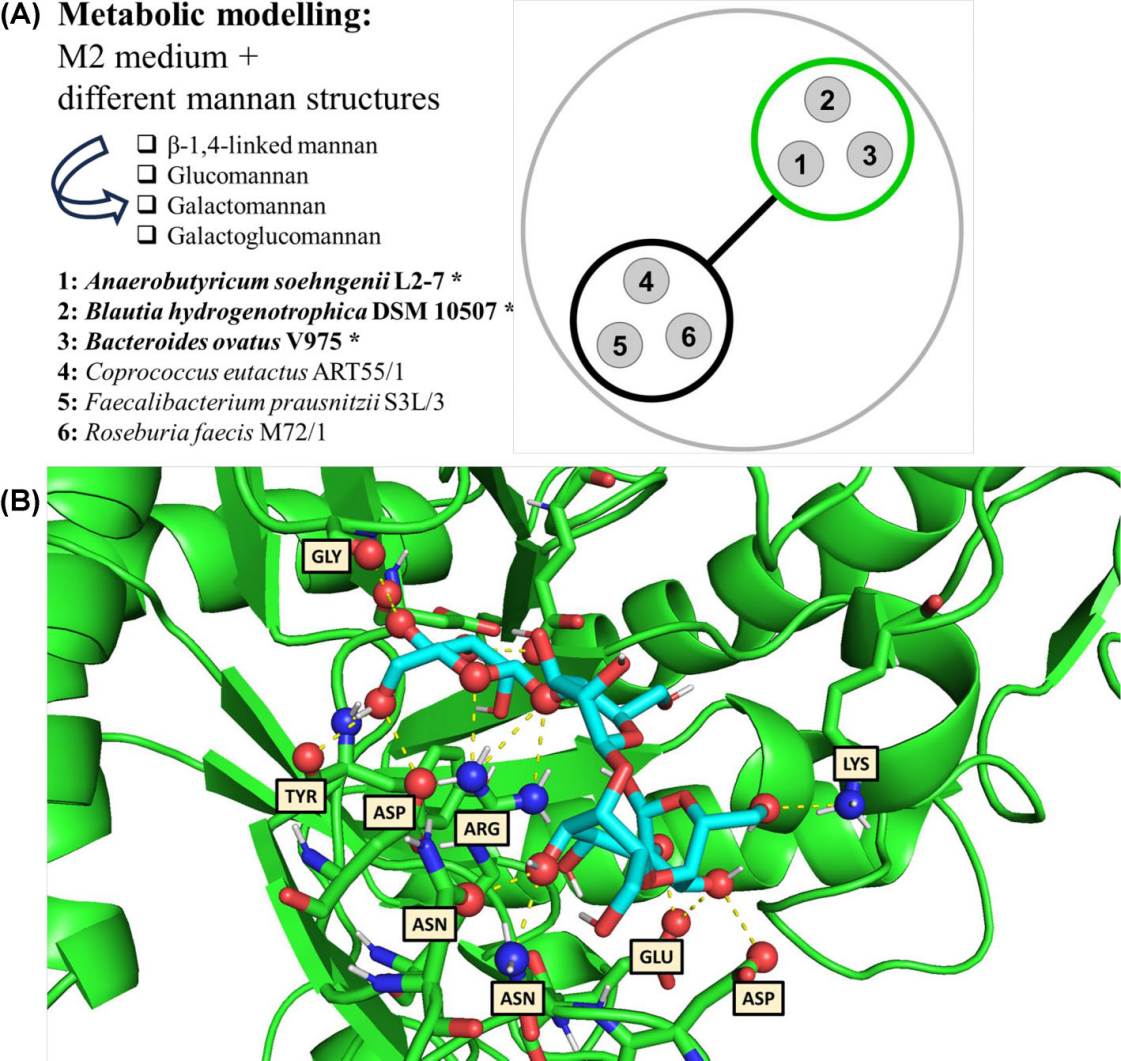
(A) Metabolic network illustrating potential cross-feeding mechanisms between microbial strains included in mixed bacterial cultures to ferment different mannan structures (β-1,4-linked mannan, glucomannan, galactomannan, galactoglucomannan). Network nodes (i. e. circles containing different microbial communities showing equivalent metabolic functions) are connected by black lines indicating synergistic relationships between communities and complementary metabolic functions. Green node corresponds to essential symbionts (i.e. key species needed to obtain all producible metabolites in the microbial community, highlighted in bold, *). (B) Potential interaction mechanisms, determined by molecular docking, between β-1,4-linked mannan structures (mannotetraose), and mannase and mannosidase enzymes from bacterial strains under study (GH26 from *Roseburia faecis* M72/1 illustrated in this figure). Polar contacts between catalytic residues from the active site and the mannan backbone are shown as dashed lines. ASN: asparagine, ASP: aspartic acid, GLN: glutamine, GLU: glutamic acid, GLY: glycine, LYS: lysine, TYR: tyrosine.

Once CAZyme profiles of bacterial isolates were determined, synergistic interactions of microbial strains included in mixed bacterial cultures were elucidated through genome-scale metabolic models (GSMMs) (Fig. 6A). Metabolic network modelling revealed that *A. soehngenii* L2-7, *B. hydrogenotrophica* DSM 10507, and *B. ovatus* V975 are essential symbionts involved in the colonic fermentation of mannan structures (β-1,4-linked mannan, glucomannan, galactomannan, and galactoglucomannan) (Fig. 6A). Essential symbionts are key micro-organisms that are required in every minimal community of microorganisms to conduct one specific function through metabolic cooperation, such as the metabolism of mannan. These essential symbionts exert unique metabolic functions in the microbial community. In this regard, *A. soehngenii* L2-7 produces butyrate from lactate, *B. hydrogenotrophica* DSM 10507 utilizes formate to produce acetic acid and *B. ovatus* V975 shows a wide range of CAZymes acting on mannan (Fig. 5) and is able to utilise galactomannan and glucomannan. In contrast, *C. eutactus* ART55/1, *F. prausnitzii* S3L/3, and *R. faecis* M72/1 were classified as alternative symbionts occurring only in some of these minimal communities of interacting microorganisms (Fig. 6A). These alternative symbionts comprise mannan-degrading bacteria and butyrate producers. Microbial metabolites that may be produced from cross-feeding mechanisms predicted by metabolic modelling include different amino acids (D-aspartate, tyrosine, and β-tyrosine). Therefore, metabolic modelling underscores the potential of three bacterial strains (*A. soehngenii* L2-7, *B. hydrogenotrophica* DSM 10507, and *B. ovatus* V975) to maximise amino acid synthesis through synergistic interactions in the human colon. The positive fold changes of mannan-degraders observed in the co-culture experiments using four and five strain combinations (Fig. 4) highlight the metabolic complementarity of essential and alternative symbionts.

To gain a better understanding of the enzymatic activities involved in mannan fermentation process, molecular modelling of microbial enzymes was performed. The affinity of key CAZy domains described above to β-1,4-linked mannan backbone was investigated by molecular docking. CAZy domains showing the highest affinity values to mannan chains included CBM23 (from *C. eutactus* ART55/1 and *R. faecis* M72/1, CBM23 is only found linked to GH26), GH26 (from *B. ovatus* V975, *C. eutactus* ART55/1, *E. siraeum* V10Sc8a, and *R. bicirculans* 80/3), and GH113 (from *R. bicirculans* 80/3) (Supplementary material Table S1). These enzymes showed docking affinity values of -7.6 ± 0.6 Kcal/mol (ranging from −6.8 to −8.6 Kcal/mol). It should be noted that a high-affinity binding is indicated by a more negative kcal/mol value, meaning stronger, more favourable interaction with values greater than −7.0 Kcal /mol often considered good to high affinity. In this regard, previous studies report strong molecular docking interactions between fungal β-mannanase and mannans, with a binding affinity of −7.8 Kcal/mol with galactomannan (Pandey and Kango 2025). Potential chemical interactions between mannan-degrading CAZymes and mannooligosaccharides (mannotetraose) are illustrated in Fig. 6B. These interactions mainly comprise hydrogen bonds between mannan chains and several amino acids from the enzyme active site including asparagine, arginine, glutamic and aspartic acid (Fig. 6B). Similar H-bonding interactions involving aspartic and glutamic acid, glycine, asparagine and tyrosine were previously reported for molecular docking simulations of mannosidases and mannotriose, glucomannan and galactomannan (Pandey and Kango 2025).

## Discussion

The ability to degrade β-mannans is likely to differ among bacteria belonging to different genera and species (Gherardini and Salyers 1987, Tailford et al. 2007, Kulcinskaja et al. 2013, Kawaguchi et al. 2014, Chung et al. 2017). The strains tested in this study were Bacillota species that are dominant in the human gut microbiota (Qin et al. 2010, Flint et al. 2012, Forster et al. 2019).

As reported previously, the presence of both endo-β1,4-mannanases and β-mannosidases are required to break down native β-mannans (Domingues et al. 2018). To relate the CAZyme profiles of the strains tested in this study to their growth data, the model proposed by La Rosa et al. (2019b) for β-mannan utilization in *R. intestinalis* L1-82 was used as a frame of reference with some adaptations. The process starts with a cell-surface bound GH26 enzyme (La Rosa et al. 2019b) or secreted GH5 endo-β1,4-mannanases breaking down the mannans without the need to first be internalized. The importance of GH26 was apparent when comparing the growth of *F. prausnitzii* S3L/3 on native and GH26-treated mannans as growth was much improved on treated konjac and carob mannans with little evidence of growth on the native mannans [Fig. 2(v) and Table 4]. The resulting β-MOS are then most likely transported into the cell via a high affinity binding protein and ATP-binding cassette (ABC) transporters. For glycosyl hydrolases to act on mannans, the β-1,4 bonds linking glucose and mannose residues in the mannan backbone have to be cleaved through the activity of endo-β-mannanases and/or endo-β-glucanases to release manno-oligosaccharides. As glucomannans and galactoglucomannans are acetylated, the presence of carbohydrate esterases would also facilitate the action of intracellular β-mannanases (La Rosa et al. 2019b, Lindstad et al. 2026), by removing acetyl groups to make the mannan backbone more accessible (Pauly and Ramírez 2018). Carbohydrate esterase CE17 was identified from *R. intestinalis* (Michalak et al. 2020b) and may therefore be more applicable to Bacillota. The β-MOS generated by the action of endo-β-mannanases may also contain galactose residues linked as side chains. Intracellular α-galactosidase would not be able to release galactose from galactomannans and galactoglucomannans until after GH26 and/or GH5 have cleaved the mannan to the appropriate chain length prior to transportation into the cell. After the decorative side-chains have been removed from the mannan backbone, then β-mannosyl-phosphorylases such as GH130 release monosaccharides from the manno-oligosaccharides (La Rosa et al. 2019b). It should be noted that GH27 α-galactosidases were missing from most of the bacterial strains under investigation in this study. This is consistent with the knowledge that GH27 α-galactosidase have so far been predominantly detected in fungal strains (Wang et al. 2025).

The growth of the nine Bacillota when supplied with various β-mannans (Fig. 2) in this study corresponded mostly very well with their CAZyme profiles, although there was little utilisation of acetylated spruce galactoglucomannan by of *F. prausnitzii* S3L/3, possibly due to decreased binding affinity of its mannan binding protein to manno-oligosaccharides that are more heavily acetylated. Indeed, previous work has showed that *R. intestinalis* L1-82 revealed a substantial decrease in binding affinity on diacetylated mannotetraose compared to mannotetraose (La Rosa et al. 2019b). As GH26 and GH5 are key endomannanases involved in the β-mannan degradation process, bacteria that lack these families, such as *A. soehngenii* L2-7*, B. hydrogenotrophica* DSM 10507, were unable to metabolise native β-mannans (as can be clearly seen from the single culture growth experiments (Fig. 2).

With respect to *Bifidobacterium* species, it should be noted that *B. adolescentis* L2-32, lacking GH26 β-mannanase, was not able to degrade β-mannans in the current study (Fig. 3). A similar observation was made where *B. adolescentis* ATCC 15703 had limited growth on carob galactomannan and konjac glucomannan (Bhattacharya et al. 2021). When investigating the CAZyme profile of *B. adolescentis* L2-32, it evidently lacked several GH5 subfamilies, GH130 as well as CEs. It has been shown previously that three mannan utilisation loci: (1) GH1, GH2, an ABC transporter; (2) GH5_8; and (3) GH36, were likely responsible for the degradation of mannans by *Bifidobacterium animalis* subsp. *lactis* ATCC 27673 (Ejby et al. 2019). The authors also highlighted the ABC transporter for β-MOS uptake because *B. animalis* subsp*. lactis* Bl-04, a strain without the transporter, was not able to utilise β-mannans despite possessing GH5 subfamily 8. This ABC transporter may be absent in *B. adolescentis* L2-32, and together with the missing CAZyme families listed above, may have led to the lack of growth on the native and GH26-treated β-mannans. This also demonstrates how β-mannan utilisation ability differs within strains of the same species, suggesting that when developing probiotics, it is essential to focus on the strain of interest.

Furthermore, this seems to also be the case for *B. ovatus*, with some strains possessing the ability to degrade galactomannans while others may not (Reddy et al. 2016). Bacteroides* ovatus* strain V975 included in our study, however, was able to utilise galactomannan and glucomannan as shown previously for *B. ovatus* ATCC 8483 (Bågenholm et al. 2017, La Rosa et al. 2019a). Intriguingly, *B. ovatus* V975 did not show detectable growth on acetylated spruce galactoglucomannan (Table 4) probably due to the lack of CEs domains. *Bacteroides* spp. possess polysaccharide utilisation loci (PULs) that contain genes encoding GH26s, GH130s, GH3s and GH36, allowing the breakdown of β-mannans (Eisen et al. 2013,, Kawaguchi et al. 2014, Bågenholm et al. 2017). In particular, the role of the exo-acting mannosidase GH2 has been highlighted as important in *B. thetaiotaomicron* (Tailford et al. 2007), which was not involved in the process used by *R. intestinalis* L1-82 (La Rosa et al. 2019b), for the degradation of undecorated manno-oligosaccharides.

The composition of GH26-treated konjac glucomannan is mostly mannose, mannobiose, mannotriose, mannotetraose, mannopentaose, mannohexaose, and mannoheptaose while pre-digested carob is mannose, mannobiose, galactosylmannotriose, digalactosylmannotetraose, galactosylmannotetraose, and digalactosylmannopentaose (Lindstad et al. 2021). It is likely that slightly poorer growth on the pre-digested mannans for *C. eutactus* ART55/1 and *B. ovatus* V975, indicate differences in mechanisms for β-mannan degradation in these two strains compared to that of *R. intestinalis*, which was used as the model in the current study. Additionally, both strains possess GH26 which may functionally differ from *Ri*GH26. Mannan residues with chain lengths different from that listed above may also be the preferred substrate for *C. eutactus* ART55/1 and *B. ovatus* V975.

It has been reported previously that *F. prausnitzii* S3L/3 was able to grow on native konjac glucomannan when co-cultured with *B. ovatus* V975 (Lindstad et al. 2021), which possesses a β-mannan PUL that includes GH26 (Bågenholm et al. 2019). The absence of enzymes, such as the aforementioned GH26, could however be circumvented by culturing strains lacking this activity with another or several that possess GH26 activity [Fig. 4B(iii)]. Furthermore, cross-feeding on breakdown products as well as other fermentation products between β-mannan degraders and beneficial non-degraders could potentially promote growth of strains lacking GH26 activity. Indeed, the growth experiments with mixed bacterial cultures showed that even when six bacterial strains (including three mannan non-degraders) were cultured together with konjac glucomannan as a carbon source, all the strains showed higher fold changes relative to that seen on M2 basal media [Fig. 4B(i) and (iii)]. The results obtained using native β-mannans as test substrates suggest that it is not necessary for the pre-processing of mannans to β-MOS in order for them to selectively stimulate beneficial members of the gut community.

Our findings show that the ability to utilise β-mannans is present in most of the Bacillota strains included in this study and these were selected as representatives of the dominant species in the human colon (Qin et al. 2010, Flint et al. 2012, Forster et al. 2019) belonging to different genera, namely *Roseburia, Eubacterium, Coprococcus*, and *Ruminococcus* (Fig. 2). The overwhelming ability of the Bacillota species in this study to degrade this glycan suggests that these strains could play a predominant role in the metabolism of β-mannans in the human gut (Figs 2 and 3).

Results from the genome mining analysis highlight the metabolic potential of these strains to metabolise the β-1,4-linked mannan backbone, of glucomannan, galactomannan and galactoglucomannan. In this regard, *R. intestinalis* L1-82, *R. faecis* M72/1, and *C. eutactus* ART55 showing CAZymes acting on β-mannan, including acetylmannan esterases, were also able to metabolize carob galactomannan, konjac glucomannan, and softwood spruce acetylated galactoglucomannan. It should be noted that some of these CAZymes showed high affinity values to mannan chains compared to other enzyme domains. In contrast, *F. prausnitzii* S3L/3 only grew well on β-mannan endo-mannanase digests, in effect due to the absence of several CAZymes acting on galactose and glucose residues compared to other bacterial strains. Molecular docking highlights the ability of these bacteria to metabolise mannan chains while metabolic modelling suggests complementary and synergistic interactions between *Bacillota* and *Bacteroidetes* β-mannan utilising strains. This may explain the capability of co-cultures performed in this work.

In conclusion, several butyrate producers belonging to the Bacillota phylum tested in this study were able to utilise carob (galactomannan), konjac (glucomannan), and spruce (acetylated galactoglucomannan) mannans as growth substrates. The prebiotic potential of β-mannans suggested here make these glycans promising candidates for future studies, and for development of symbiotic formulations that include beneficial butyrate producers. In particular, spruce-derived mannans offered the greatest selectivity, mainly promoting growth of the Bacillota strains, as previously demonstrated in weaning pigs (Michalak et al. 2020a). Spruce acetylated galactoglucomannan could therefore offer a promising solution to valorise forestry waste as food additives to promote health and reduce the use of additives that may be detrimental to health (Chassaing et al. 2017, Naimi et al. 2021).

## Supplementary Material

fiag063_Supplemental_Files
