# Supplementary material for: Metabolism of β-mannans by representative, understudied Bacillota species from the human colon
Source: FEMS Microbiol Ecol. 2026 Jun 12;102(7):fiag063. doi: 10.1093/femsec/fiag063 (PMC13278490; doi:10.1093/femsec/fiag063)
Supplement: fiag063_Supplemental_Files [file fiag063_supplemental_files.zip › Supplementary_table (1).docx]

**Table S1**. Top 10 Carbohydrate-Active enZYmes (CAZy) showing the highest molecular docking affinities (Kcal/mol) to β-1,4-linked mannan structures (degree of polymerization 4). These enzymes were annotated in the genome sequences of bacterial strains under study (see **Figure 5**) and involve mannase and mannosidase activities. Potential chemical interactions are illustrated in **Figure 6**.

| **Organism** | **CAZy** | **Enzyme number** | **Kcal/mol** |
| --- | --- | --- | --- |
| *Coprococcus eutactus* ART55/1 | CBM23 | 1 | -8.6 |
| *Coprococcus eutactus* ART55/1 | GH26 | 1 | -7.5 |
| *Coprococcus eutactus* ART55/1 | GH26 | 2 | -7.1 |
| *Eubacterium siraeum* V10Sc8a | GH26 | 1 | -6.8 |
| *Ruminococcus bicirculans* 80/3 | GH26 | 1 | -7.6 |
| *Ruminococcus bicirculans* 80/3 | GH113 | 1 | -8.3 |
| *Roseburia faecis* M72/1 | GH26 | 1 | -7.7 |
| *Roseburia faecis* M72/1 | CBM23 | 1 | -7.5 |
| *Bacteroides ovatus* V975 | GH26 | 1 | -7.9 |
| *Bacteroides ovatus* V975 | GH26 | 1 | -6.8 |
