# Supplementary figures and images for: Metabolism of β-mannans by representative, understudied Bacillota species from the human colon
Source: FEMS Microbiol Ecol. 2026 Jun 12;102(7):fiag063. doi: 10.1093/femsec/fiag063 (PMC13278490; doi:10.1093/femsec/fiag063)

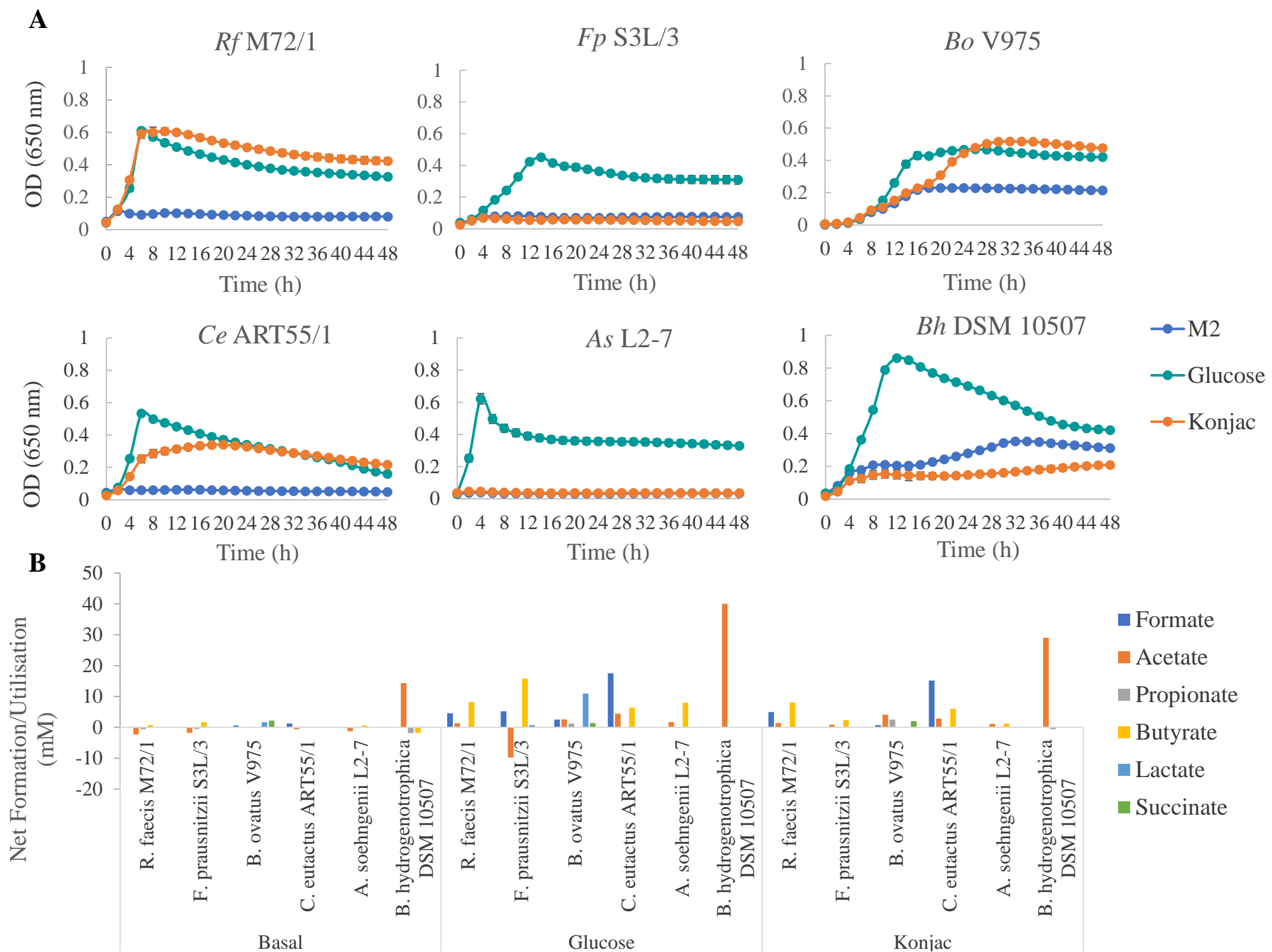

Supplemental Figure 1R

Supplement: fiag063_Supplemental_Files [file fiag063_supplemental_files.zip › Figure_S1.pdf]
